# The Use of Artificial Intelligence in Improving Diagnostic Modalities in Rheumatoid Arthritis: A Narrative Review

**DOI:** 10.7759/cureus.102992

**Published:** 2026-02-04

**Authors:** Sara Tariq, Arshia Ahmed, Gurdeep Singh, Paul Dura

**Affiliations:** 1 Internal Medicine, Guthrie Lourdes Hospital, Binghamton, USA; 2 Endocrinology, Diabetes and Metabolism, Guthrie Lourdes Hospital, Binghamton, USA; 3 Rheumatology, Guthrie Lourdes Hospital, Binghamton, USA

**Keywords:** artificial intelligence, diagnostic modalities, machine learning models, natural language processing models, rheumatoid arthritis

## Abstract

Rheumatoid arthritis (RA) is an inflammatory autoimmune condition affecting the joints and other organs such as the heart, eyes, and lungs. For decades, it has been diagnosed through assessing a combination of clinical picture, serologic biomarkers, and radiographic studies. However, the possibility of false negative test results and inability to detect early arthritic changes make RA diagnosis challenging. The diagnostic accuracy of the RA diagnostic modalities has substantially improved since the emergence of artificial intelligence (AI)-based medical algorithms, resulting in timely disease prediction and prevention of irreversible joint damage. AI computational models employ machine learning (ML), natural language processing (NLP), and rule-based expert systems to enhance the diagnostic accuracy of rheumatological diseases, particularly rheumatoid arthritis. AI-based algorithms not only identify specific disease patterns to predict the early course of disease but also use visual scoring systems, enhancing imaging characteristics. Radiological studies such as X-ray, MRI, CT, and PET scan can quantify joint space narrowing, cartilage loss, synovitis, bone erosions, and bone marrow edema. In addition, ML-integrated microRNA gene profiling reshaped the microenvironment of joint space by modulating gene expression and reducing joint deterioration in rheumatoid arthritis patients, surpassing the rheumatoid factor (RF) and cyclic citrullinated peptide (CCP) risk scoring models.

## Introduction and background

Artificial intelligence (AI) possesses the capability of performing human tasks using computer systems by employing human intelligence, such as planning and decision-making, social intelligence, perception, natural language processing (NLP), learning, reasoning, and problem-solving [1]. In the future, AI algorithms may examine patient information, medical background, and symptoms by identifying patterns in enormous patient data, which may be valuable in diagnosing diseases through early prediction and prevention before symptom development. It will add potential for predictive medicine to grow; however, ethics, data interpretability, and quality need to be addressed [2].

Rheumatoid arthritis (RA) is a chronic, systemic inflammatory autoimmune disease characterized by painful, swollen joints, which significantly impact the quality of life of patients [3]. It is one of the most common rheumatological ailments, causing substantial functional disability in huge patient populations [4]. The diagnosis of RA remains challenging despite significant advancements in diagnostic modalities, as images may not always detect disease activity and may result in possible false negative results [5]. However, AI can be effective in identifying the inflammatory signs of RA in its early stages by recognizing specific disease patterns [6]. AI has significantly revolutionized the diagnostic modalities for RA patients by overcoming diagnostic delays [5].

This article aims to describe the role of AI in rheumatology, with a special focus on RA. This literature review also highlights the use of AI in enhancing diagnostic modalities in RA, detailing its benefits, challenges, and limitations. It explains how AI identifies specific disease patterns by quantifying joint space narrowing, cartilage loss, bone erosions, synovitis, bone marrow edema, and reduces joint deterioration by modulating gene expression.

## Review

Methods

A comprehensive literature search was conducted across multiple databases including PubMed and Google Scholar from 2000 to August 2025 using the MeSH terms 'Rheumatoid Arthritis diagnosis and Artificial Intelligence', including full text in English and Spanish from article types (clinical trials, systematic review, scoping review, meta-analysis, books/documents), excluding preprints and editorials, and including males and females of all age groups, to study the enhancements in diagnostic standards in RA through the emerging world of AI-based medical technologies. No quality risk appraisal was performed.

Artificial intelligence 

In general, AI started with the invention of robots, influenced by da Vinci's sketchbooks of robots. The term is derived from the Czech word *robota* and was officially coined in 1956. It is also described as the science of developing intelligent machines, largely applicable to medical diagnosis and statistics [7]. 

AI makes and implements different methods and software to make intellectual decisions pertaining to its environment [1]. It can analyze and understand external data to achieve desired goals [8]. AI is reshaping the world through the emergence of ChatGPT (OpenAI, San Francisco, CA, USA) and Google's Gemini (Mountain View, CA, USA), influencing millions; however, regulatory and mental health concerns have also risen [9]. 

AI research revolves around reaching certain goals by using different techniques and methods. Mostly, the techniques used are formal logic, artificial neural networks, and mathematical optimization, whereas the methods involve economics, statistics, and operations research [10]. In addition to the general applications, AI also carries the potential to improve the quality of life of patients with better health care through its applications in medicine and medical research [11]. 

AI has become an essential tool for medical research. It helps with processing and integrating large and complex datasets, for example, using a microscopic imaging technique for tissue engineering development [12]. With new advancements in AI tools, there is a better understanding of biomedical pathways. Examples include AlphaFold2 from 2021, which created a 3D protein structure in hours [13], a new class of antibiotics susceptible to two different types of bacteria was discovered in 2023 through AI-guided drug discovery [14], and Parkinson's disease screening process was accelerated with the development of potential drug treatments using machine learning (ML) in 2024 [15,16]. 

**Figure 1:**
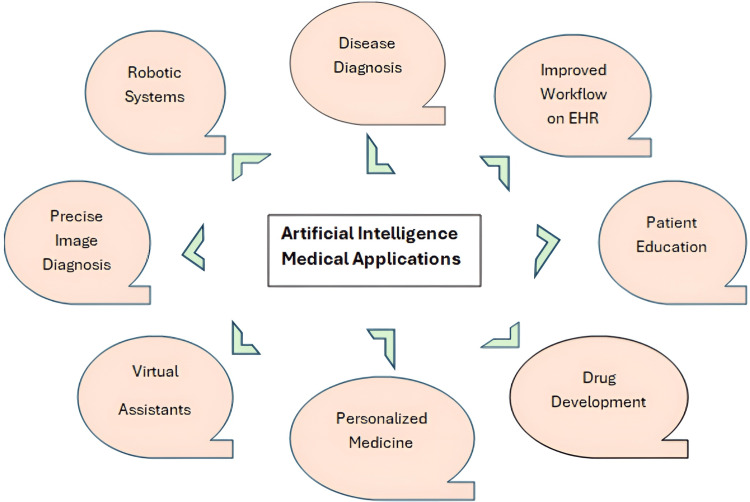
Artificial Intelligence Medical Applications EHR: Electronic Health Record

AI applications in healthcare are intended to provide quicker ways to prevent, diagnose, and treat medical diseases by analyzing complex data from medical health systems [17,18]. AI medical applications are applied in diagnostic modalities [19], development of treatment protocols [20], development of drugs [21], patient monitoring and care [22], and personalized medicine [23]. AI-related medical applications are displayed in Figure 1. 

Technologies used by AI relevant to healthcare include ML, natural language processing, rule-based expert systems, physical robots, and robotic automation processes [11]. The details of further applications used and the previously mentioned techniques are displayed in Table 1 [11]. 

**Table 1:** AI technologies and applications used in medical diagnostic modalities

AI technologies used in medical diagnostic modalities	Applications
Machine learning	Precision medicine, neural networks, deep learning (Large Language Models).
Natural language processing	Creation, understanding, and classification of clinical documentation and published research.
Rule-based expert systems	Clinical decision support rules set by human experts and knowledge engineers.
Physical robots	Perform predefined tasks in healthcare, including surgical Robots to assist in minimally invasive incisions and stitch wounds.
Robotic process of automation	Computer programs on servers used for repetitive tasks like prior authorization, updating patient records of billing.

This review will focus on the use of AI applications in enhancing diagnostic modalities of RA with a brief introduction to the general use of AI in diagnosing diseases.

General Use of AI in Diagnosing Diseases 

AI has the data processing capabilities to help in accurate diagnosis by saving time and improving healthcare outcomes through enhanced accuracy [24]. AI reviews mass medical health records through the use of ML to aid in the diagnosis of different ailments for patients [25]. AI also applies its medical applications for the diagnosis and treatment of diseases. For example, IBM's Watson (Yorktown, NY, USA) was known for using precision medicine for cancer diagnosis and treatment [11]. It helps in early disease prediction through identifying high-risk individuals [26]. AI uses decision support systems to help prioritize and triage patients with serious cases in urgent scenarios such as the Emergency Department [24].

A cross-sectional study from 2023 compared the satisfaction rates of responses given to patient questions on the public platform of Reddit’s r/AskDocs. It showed higher satisfaction with ChatGPT-generated responses compared to clinicians, which highlights the potential effectiveness of AI assistants in patient messaging workflows to improve efficiency [27]. However, some authors argue that the coauthors evaluated the experiment, and it can lead to decreased accuracy of the experiment due to not using a blinding study protocol [28]. 

Use of AI in Rheumatology 

An AI-based cognitive virtual assistant was launched by Versus arthritis (Chesterfield, United Kingdom) in collaboration with IBM Watson to assist patients with rheumatological illnesses by addressing their concerns and triaging for consultation. This helps in better control and management of chronic rheumatological diseases through long-term follow-up [29]. 

Rheumatological diseases evolve over time and are chronic in nature. It helps rheumatologists to summarize relevant information from patients' medical records and reduces the time spent on analyzing patient records [29]. 

AI also creates plugins, meaning it incorporates software into existing applications. This technology is employed in RBknee (Radiobotics Copenhagen, Denmark), an FDA-approved device, which helps in the assessment of radiographic changes in osteoarthritis [29,30]. 

Vision Transformer (ViT) helps rheumatologists in developing high-quality nailfold capillaroscopic (NFC) reports by assessing the patterns of microangiopathy to help in systemic sclerosis screening and diagnosis [29,31]. Garaiman A et al. also compared the ViT's analysis performance with rheumatologists, concluding that the final diagnosis of a scleroderma pattern still is dependent on an experienced observer [31].

Use of AI in Diagnosing RA

AI offers huge benefits in rheumatology by improving the efficiency in healthcare, reducing the variability and limiting medical errors. It also streamlines medical care by assisting physicians in making clinical decisions [32]. 

Rule-based algorithms using automated image recognition methods are the most advanced in predicting the course of RA [33]. 

Current standards for diagnosing RA combine the clinical picture, serological evidence of biomarkers like rheumatoid factor (RF) and cyclic citrullinated peptide (CCP), and radiographic studies like X-rays, computed tomography (CT) scan and magnetic resonance imaging (MRI). However, there are limitations due to the possibility of false-negative serologic testing and delayed findings of activity of early RA on imaging. Moreover, current diagnostic protocols are time-consuming, which can lead to irreversible joint damage and impaired quality of life [5]. 

AI offers a revolution in the management and monitoring of RA patients by providing more accurate diagnoses and personalized treatment options [5]. AI shows promising results in early diagnosis of RA by utilizing ML-based algorithms to study substantial datasets pertaining to clinical scenarios and imaging [5]. It also quantifies synovitis through MRI and ultrasound imaging to assess the severity of RA [34].

Diagnostic modalities 

Radiographic Studies 

AI has the potential to aid in the interpretation of radiological imaging [35]. It deciphers medical imaging such as X-rays, MRI, and CT scans to improve diagnostic precision [2]. It employs radiomics and computer vision in radiological studies to characterize the features in a joint space, like edges, texture, densities, and spatial relationships [36]. 

On one hand, radiologists, in general, have a short time to comment on radiological images. When the workload increases, there is a chance of an increase in medical errors, too. But on the other hand, there are technical limitations on the accuracy of studies as well as the influence of image artifacts and variations in normal anatomy. This, in turn, raises a question about AI positively impacting the efficiency and efficacy of healthcare [37]. 

X-ray 

AI-assisted Chest X-ray uses the technique of deep learning to identify patterns based on features in large data sets. It helps in identifying the patterns and reporting delays by radiologist by decreasing their workload [38]. 

Phatak et al. suggested that the diagnostic accuracy of inflammatory hand arthritis improves with the potential use of AI computational systems such as convolutional neural networks (CNNs) on standardized smartphone images. It helped in detecting inflammation in the wrist, proximal interphalangeal (PIP) 2, and PIP 3 joints of patients with various rheumatic diseases. Their study involved an Indian patient cohort where patients with early inflammatory arthritis and healthy cohorts were examined first by two rheumatologists and then pre-trained CNN models fine-tuned with a preset data set were used to analyze standardized hand photographs, anonymized and cropped around joints. The study analyzed 800 hands, including 200 controls and 200 patients with early inflammatory arthritis, with the wrist being the most commonly involved joint (173/400), followed by the middle finger proximal interphalangeal joint (MFPIP) (134) and the index finger proximal interphalangeal joint (IFPIP) (128). The results showed 0.89 concordance between two rheumatologists and the CNN achieved 99% accuracy and specificity and 98% sensitivity in predicting a patient with early inflammatory hand arthritis, showing great promise as a screening tool. However, future research requires generalizability on diverse patient populations and involvement of other joints [39]. 

Signs of inflammation like bone erosions are identified with more accuracy in X-ray and MRI studies by employing CNN technology. A study conducted in 2022 demonstrated that an AI-based model analyzed a dataset of 10,000 digital X-rays achieving a sensitivity of 94% and specificity of 91% for diagnosing bone erosions [5]. 

Plain X-rays can automatedly quantify cartilage loss in RA patients indirectly through measurement of radiographic wrist, metacarpophalangeal (MCP), or PIP joint space width as cartilage loss cannot be seen directly on X-rays. Likewise, bone erosions on X-rays can be analyzed using bone contour details through automation technology [36].

Currently, the modified Total Sharp Score (mTSS) is a promising tool to quantify joint damage for assessing disease progression in RA patients. However, it was tedious and time-consuming highlighting the need for an efficient and improved version of mTSS. Consequently, Wang HJ et al. not only proposed an automatic labeling system by detecting the joint space narrowing on hand X-rays leveraging the You Only Look Once (YOLO) model, but they also developed a joint classification model in recognizing mild RA disease, suggesting improved outcomes and accuracy if one hand X-ray protocol is incorporated in the mTSS model [40]. 

MRI Analysis 

AI analyzes subtle changes in radiographic MRI using visual scoring systems and detects inflammation, which assists in early diagnosis of RA [6].

AI-based ML algorithms help in analyzing and visualizing inflammation in rheumatic diseases using advanced imaging techniques such as fluorescence optical imaging (FOI) [32].

A review article from 2023 by Nicoara AI et al. highlights the integration of AI for the diagnosis of RA through magnetic resonance imaging [41]. In addition, another review by Gilvaz and Reginato also emphasizes the use of AI and optimization of management strategies for RA, including diagnosis and treatment [42].

The standard manual radiographic technique for assessing RA-related lesions on MRI was Rheumatoid Arthritis Magnetic Resonance Imaging Scoring (RAMRIS) which was employed after acquisition of MRI [41]. RA-related abnormalities assessed by the RAMRIS system include synovitis, bony erosions, and bone edema of the wrist joints to aid in diagnosis [34]. Czaplicka K et al. presented an automated method by determining inflamed synovial membrane volume based on the pre- and post-contrast MR images and wrist bones segmentation analysis. They found that the RAMRIS score for manually quantified synovitis volume was in the range of 0.75-0.81, and the computational quantified synovitis volume was in the range of 0.76-0.87 for the total RAMRIS synovitis score. It demonstrated great potential of automated assisted methods for assessment of synovitis [34].

The most sensitive method to quantify bone marrow edema and periarticular inflammation, in addition to subchondral bone erosions and ankylosis, is MRI compared to low-dose CT scan [41]. Aizenberg et al. evaluated an automated technique to quantify bone marrow edema (BME) to detect early RA changes on MRI as the standard method requires the scoring of 61 joint-level features. On comparison with visual assessment, the automated approach proved to be a robust alternative for assessment of BME in the RA patient population without compromising the accuracy of prediction of RA and reducing the scoring efforts of MRI analysis [43].

Ultrasound 

AI-assisted deep learning models do the segmentation of structures and ultrasound images to aid in the interpretation. For example, some researchers formulated an AI prediction model to identify and score osteophytes by using a validated semiquantitative method for ultrasound imaging [32].

The point-of-care ultrasound (POCUS) has emerged as a promising tool to diagnose RA. POCUS detects synovitis and cartilage damage using AI-based CNNs to automatically score the Doppler images using the 4-class OMERACT-EULAR Synovitis Scoring (OESS) system, scored from 0-3 where 0-1 indicates healthy and 2-3 indicates disease [42].

AI can also assist in classifying and differentiating inflammatory arthritis, like RA, from other arthritis by looking at the 3D joint shape patterns using neural network-based deep learning models in ultrasound [44].

The MEDUSA project was developed in 2016 using a computer-aided diagnostic system which included a prototype and algorithms for analyzing the severity of synovitis in RA patients. It used semiquantitative ultrasound with power Doppler for assessing synovitis with the Medusa prototype, focusing on bone, joint, and synovitis detectors [4].

CT Scan 

High-resolution peripheral quantitative CT uses scoring based on imaging to help analyze MCP joints, which could help in differentiating arthritis from normal joints [29,45].

Topfer D et al. developed a new precise three-dimensional (3D) segmentation technique for quantifying bony erosions based on shape, volume, and surface area of the erosion using high-resolution peripheral quantitative CT (HR-pQCT) datasets. Forty-three erosions were detected in 18 datasets with 5.66%/0.49 mm and 7.76%/0.76 mm intra-and interoperative precisions for erosion volume, respectively. This assessment assisted in diagnosing RA [45].

A study from May 2025 quantitatively assessed CT scans on two cohorts of RA-interstitial lung disease (ILD) patients using a data-driven texture analysis method (DTA). At baseline, the quantification of lung fibrosis on CT scan showed worse lung function and increased subsequent DTA scores proposed increased mortality. They recommended quantitative CT scan as a robust clinical outcome tool in RA-associated ILD [46]. 

PET Scan 

AI employs deep learning technologies for generating PET images [47]. [18F] fluorodeoxyglucose (FDG) PET/CT scan can be used to evaluate inflammation in joints by assessing synovial membrane metabolic activity through standard uptake protocol or visually [48]. 

On the other hand, fluorine-18-labeled sodium fluoride (NaF) PET/CT assesses the osteoblastic activity in synovium. These imaging techniques can be used for predicting RA severity. FDG PET/CT scan evaluates vasculitis in RA patients [49]. FDG PET/CT and NaF PET/CT scans aid in diagnosing RA early by timely identification [48]. However, more evidence is required to incorporate it in standard RA clinical management [48]. 

A pilot exploratory study in October 2024 demonstrated that [18F]AlF-FAPI-RGD PET/CT is an excellent tool in assessing disease activity in RA. It uses a dual-targeted heterodimer tracer focusing on fibroblast-activated protein (FAP) and integrin αvβ3 through PET/CT radiography [50]. 

Biomarkers 

AI guides in risk scoring of RA patients using RF and anti-CCP (ACPA) positivity, but such models do not have a good predictive value [29]. RF and CCP lack the sensitivity and specificity, which results in a delay in diagnosing RA. Recently, AI and ML-integrated algorithmic microRNA profiling has emerged as a favorable phenomenon for revolutionizing the diagnosis and treatment of RA. Specifically, miR-126 and miR-24 shape the microenvironment of RA by modulating gene expression and miR-146a and miR-155 reduce joint deterioration [51].

Geng L et al. conducted the first study to analyze and compare the effectiveness of single and combined autoantibodies in RA diagnosis guided by a sensitive neural network model. The neural network model performed better than the single and combined antibody detection, with a sensitivity and specificity up to 0.90 and 0.86, respectively [52].

ML-based algorithms in combination with biostatistical analysis provide meaningful insight into diagnosing RA-ILD patients and recognized KL-6, d-dimer, and tumor markers as potential biomarkers associated with RA-ILD patients [53].

**Figure 2:**
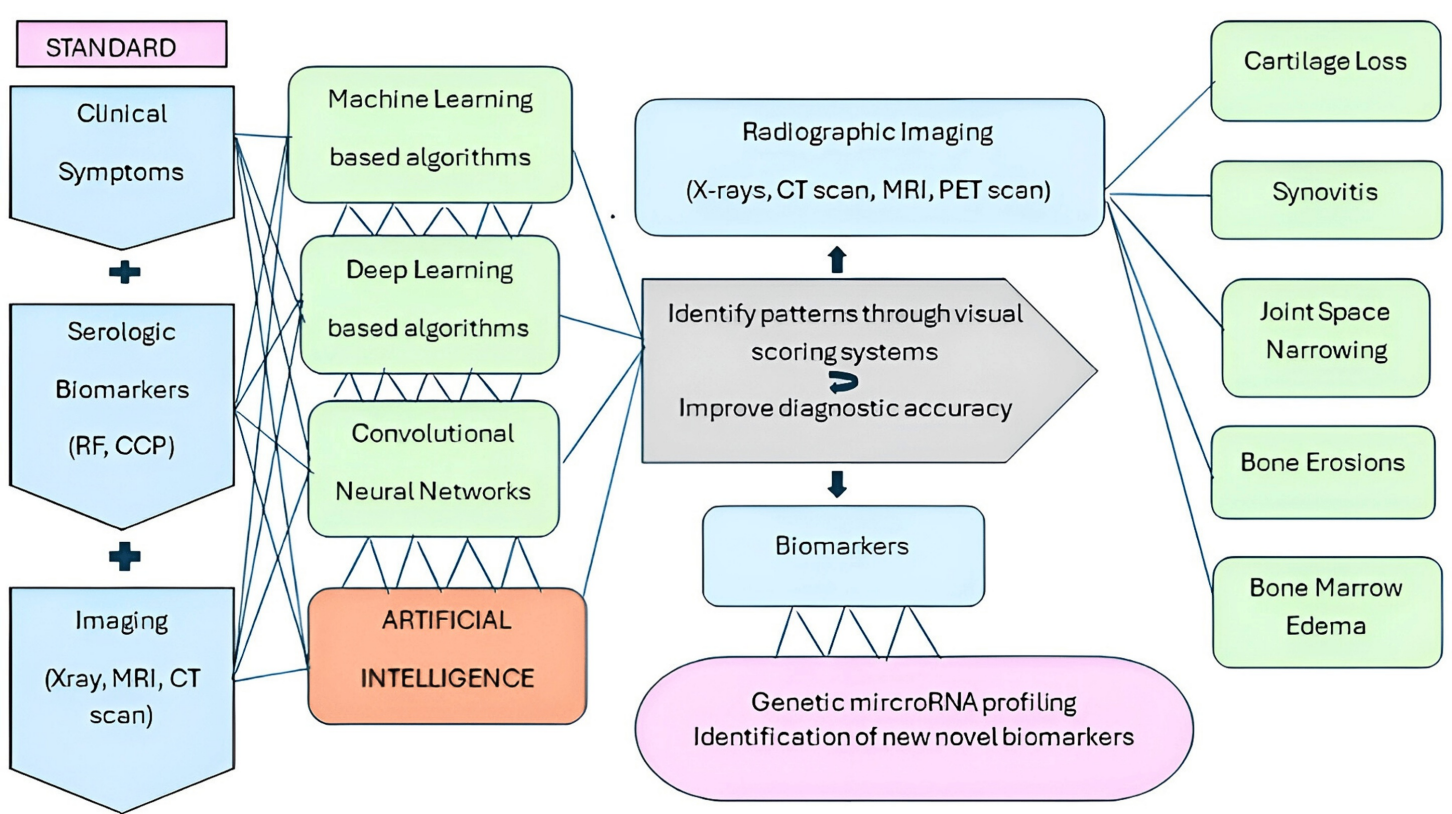
How AI improves the diagnostic modalities of rheumatoid arthritis compared to the standard protocol RF: rheumatoid factor; CCP: cyclic citrullinated peptide; MRI: magnetic resonance imaging; CT scan: computed tomography scan; PET scan: positron emission tomography

Standard rule-based RA algorithms incorporate disease-modifying antirheumatic drugs (DMARDs), codes and CCP or use eMERGE (genomics) algorithm for diagnosing RA. A retrospective study at Mayo Clinic in February 2025 by Kronzer VL et al. demonstrates that the incorporation of additional variables like BMI, smoking status, and self-reported RA through biobank-driven data improves the diagnostic efficacy in RA patients by outperforming standard RA algorithms [54]. The summary of the AI-incorporated algorithms improving the diagnostic modalities for RA is presented in Figure 2. 

Limitations/risks and mitigation strategies

On one hand, AI provides benefits but on the other hand, it raises some potential risks. It might result in loss of accountability as physicians will rely on AI-driven disease diagnoses, though they should use their own clinical judgement in accepting medical expert systems' recommendations [1]. AI-based solutions for medical disease diagnosis also requires reliability of software and hardware which can be achieved by enhancing the power and sensing capabilities of designs employed in smartphones along with a system on chip (SoC) [55]. It raises concerns regarding privacy including access to personally identifiable data [56] and copyright infringement accusations which can be rectified by creating licensed databases [57]. There is also a question of transparency into how the AI systems work to reach complex decisions as it is difficult to challenge them, trace their decisions and remove ingrained biases [58]. Hence, there is a need for auditable and comprehensive algorithms to guarantee the transparency of AI-guided recommendations in the future to enhance security [33].

In addition, it can amplify algorithmic bias by creating unfair outcomes in computerized systems [59] and can also pose some technical limitations and regulatory hurdles [32]. Moreover, AI delivers critical and pertinent patient information without any human emotions like empathy [29].

Nonetheless, there are concerns about costs and the use of resources for training staff to use AI-based technologies in rheumatology. More importantly, physicians and patients show reluctance in embracing AI technologies. Therefore, an interdisciplinary approach and incorporation of educational programs to enhance familiarity with AI are recommended to adopt it in the rheumatology scope of practice [5].

Despite significant advancements in achieving higher accuracy in diagnosing rheumatological diseases, the FDA has not approved a single AI-based tool in rheumatology as of early 2025. This fact also enlightens the importance of an interdisciplinary framework to address the clinical complexity of rheumatological diseases [60]. 

Qualitative results

In summary, the reviewed literature highlights the great potential of AI to transform rheumatology through improved diagnostic accuracy, automation of scoring systems, and biomarker discovery. However, successful integration into clinical practice will require addressing concerns around transparency, bias, cost, regulatory oversight, and user acceptance by employing mitigation strategies.

## Conclusions

AI-based algorithmic models have revolutionized the conventional diagnostic protocol for RA by preventing delays in RA recognition through enhanced diagnostic applications. Early arthritic inflammatory changes can be identified quickly through AI-integrated technologies, which can be missed by the human eye, resulting in the timely recognition of RA flares. It also helps in assessing the severity of the disease, providing accurate diagnoses, and formulating personalized treatment plans, ultimately leading to an improvement in the quality of life for patients. Therefore, developing a multidisciplinary framework and conducting educational programs are recommended to enhance familiarity with rheumatological AI applications and address the roadblocks in incorporating AI tools in rheumatology. 
